# Silicon Nanotubes as Potential Therapeutic Platforms

**DOI:** 10.3390/pharmaceutics11110571

**Published:** 2019-11-01

**Authors:** Nguyen T. Le, Yuan Tian, Roberto Gonzalez-Rodriguez, Jeffery L. Coffer

**Affiliations:** Department of Chemistry and Biochemistry, Texas Christian University, Fort Worth, TX 76129, USA; nguyen.t.le@tcu.edu (N.T.L.); Yuan.tian@tcu.edu (Y.T.); R.gonzalezrodriguez@tcu.edu (R.G.-R.)

**Keywords:** silicon nanotubes, surface chemistry, drug delivery

## Abstract

Silicon nanotubes (SiNTs) with unique well-defined structural morphologies have been successfully fabricated and recognized as a novel architecture in the nanoscale Si family. While the typical dendritic microstructure of mesoporous silicon prepared anodically has been exploited previously for therapeutics and biosensing, our status of utilizing SiNTs in this regard is still in its infancy. In this review, we focus on the fundamental properties of such nanotubes relevant to therapeutic applications, beginning with a description of our ability to sensitively tune the structure of a given SiNT through synthetic control and the associated detailed in vitro dissolution behavior (reflecting biodegradability). Emphasis is also placed here on the range of functional moieties available to attach to the surface of SiNTs through a summary of current studies involving surface functionalization and strategies that facilitate conjugation with molecules of interest for multiple purposes, including cell labeling, nucleotide attachment, and scaffolding of therapeutic metallic nanoparticles. Experiments addressing our ability to load the interior of a given nanotube with species capable of providing magnetic field-assisted drug delivery are also briefly described. Given the range of diverse properties demonstrated to date, we believe the future to be quite promising for employing SiNTs as therapeutic platforms.

## 1. Introduction

For some time, porous silicon (pSi) has attracted great attention in applications relevant to diagnosis and therapy, owing in part to its biocompatibility and biodegradability in aqueous physiologically-relevant environments [1,2,3,4]. Such a response in vitro/in vivo of pSi is sensitively dictated by porous morphology, associated Si domain dimension and surface chemistry [4,5]. While demonstrating utility in applications as diverse as bioimaging [6], drug delivery [7], and nucleotide sensing [8], pSi in a mesoporous form also exhibits some detrimental properties, namely intricate dendritric morphologies, and requires corrosive reagents in its preparation and expensive starting material (wafer grade Si). Among alternative nanostructured forms that minimize such undesirable properties, one-dimensional nanotube constructs with unique well-defined hollow interior spaces and curved side walls have captured significant interest in the investigation of new properties and potential merit in diverse fields [9,10]. To successfully prepare such a morphology, a ZnO sacrificial template method was successfully developed, which yields a broad library of silicon nanotubes (SiNTs) with controllable structural parameters (inner diameter, shell thickness, length and surface morphology) [11]; under selected fabrication conditions, porous sidewalls can also be incorporated as a part of the nanostructure morphology (pSiNTs).

While SiNTs have been actively evaluated in several applications, including Li ion batteries [12] and photovoltaics [13,14], this review focuses on biomaterial aspects of SiNTs. To be qualified as a relevant candidate in biomedical applications (e.g., drug delivery and biosensing), an understanding of stability and degradation rate of a selected matrix is required [15]. In this discussion, dissolution behavior of a large family of SiNTs at physiological temperature is emphasized, thereby elucidating biodegradability properties of a given nanotube type. In terms of therapeutic platforms, there are ample opportunities to exploit this tubular nanostructure for multiple purposes. While the inner void spaces of SiNTs are favorable for housing therapeutic species, the tunable surface chemistry of SiNTs is exploited to facilitate coupling reactions with various targeting molecules or therapeutic moieties [10]. Specifically, owing to high surface area and synthetic route, SiNTs present an oxide-rich interface; therefore, such a native oxide of SiNTs allows facile surface functionalization via formation of a stable siloxane Si-O-Si bond with a molecule containing silanol groups [16]. A well-established approach to extend functionality of the material is to use a linker with a free moiety on the distal end that can interact with molecules in the surroundings [17]. To probe the utility of SiNTs as a possible therapeutic matrix, our group has explored multiple strategies using aminosilane species, particularly 3-aminopropyltriethoxysilane (APTES), to allow conjugation to several molecules of interest, thereby: (1) Altering dispersion properties of SiNTs in aqueous environments; (2) enabling fluorescent labeling for detecting the nanotube in biological environments; and (3) facilitating complex formation with polynucleotides (e.g., plasmid DNA or siRNA) for potential gene therapy.

Thus, in this article we focus on several fundamental aspects of SiNTs relative to their possible utility as a therapeutic platform: (1) Convenient synthetic protocols; (2) temporal degradation in biologically-relevant media; and (3) surface modification strategies. As we will see shortly, the latter category has implications not only with regard to imaging and delivery, but also in our ability to create more sophisticated metal-semiconductor nanostructures, also of therapeutic value. Finally, we also demonstrate proof of concept in loading the large interior of the nanotube, utilizing superparamagnetic nanoparticles.

## 2. Fabrication of Silicon Nanotubes

**Figure 1:**
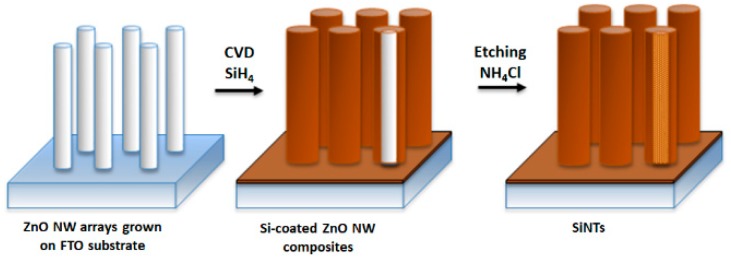
Fabrication scheme of silicon nanotubes (SiNTs)—ZnO nanowires (NWs) sacrificial template method.

**Figure 2:**
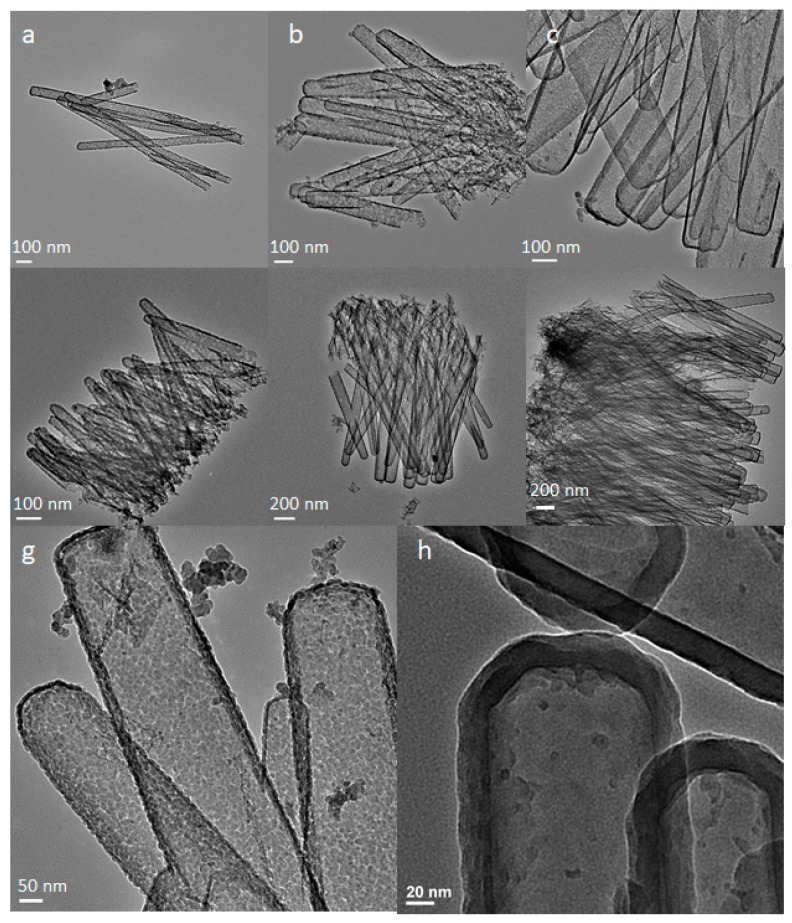
Structural and surface morphology of SiNTs. Tunable inner diameter: (**a**) 50 nm, (**b**) 100 nm, (**c**) 150–200 nm. Tunable length: (**d**) 500 nm, (**e**) 1.5–2.0 μm, (**f**) 2.5–3.0 μm. Surface morphology: (**g**) 10–12 nm highly porous side wall, (**h**) 20 nm non-porous Si shell.

Silicon nanotubes with a well-defined structure are readily fabricated via a sacrificial ZnO nanowires (NWs) template method (Figure 1) [11]. In this approach, ZnO NWs are grown on a substrate (e.g., Si wafer or fluorine-doped tin oxide (FTO) glass) pre-deposited with ZnO nanocrystals and are subsequently coated with a Si layer by performing chemical vapor deposition (CVD), in which silane (SiH_4_) diluted in He gas (0.5%) serves as a precursor. Hollow SiNTs are achieved via a gas-phase etching process, which involves decomposition of NH_4_Cl into NH_3_ and HCl at 450 ^o^C in He to remove the ZnO NW core. By controlling ZnO growth conditions (i.e., concentrations of ZnO precursors (Zn(NO_3_)_2_ and hexamethylenetetramine (HMTA) and growth time), the subsequent average inner diameter and length of SiNTs can be manipulated from 30 to 200 nm and 500 nm to 10 μm, respectively (Figure 2). Interestingly, via control of the CVD process, not only can the thickness of the Si shell can be sensitively adjusted (10–100 nm thickness), but a distinct surface morphology of SiNTs is also achieved. Specifically, when the Si sidewall thickness is limited to 10–12 nm, a unique porous morphology is obtained as a result of Si island formation via an Ostwald coalescence process. The porosity of such walls can be readily demonstrated in a simple chemical diffusion experiment involving infiltration of small luminescent molecules (e.g., the luminescent dye tris(bipyridyl) ruthenium(II), Ru(bpy)_3_^2+^) (Figure 2g) [11]. As the shell grows thicker, porous features disappear and the outer surface becomes smoother (Figure 2h). An additional annealing step at 600 ℃ in He can be performed to enhance the crystallinity of SiNTs.

## 3. Dissolution Properties of Silicon Nanotubes

**Figure 3:**
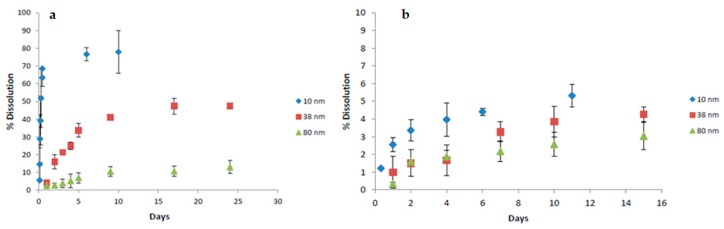
Percent dissolution of unannealed SiNTs (**a**) and annealed SiNTs (**b**) with wall thickness of 10 nm, 38 nm, and 80 nm in phosphate-buffered saline (PBS) at 37 ^o^C (adapted from [19] with permission).

Biological applications of SiNTs require careful evaluation of the biodegradation behavior of the materials in a simulated physiological environment. Based on a molybdate-based spectrophotometric method, dissolution kinetics of a variety of SiNTs have been examined [11,18,19]. In phosphate-buffered saline (PBS) at physiological temperature (37 ℃), dissolution kinetics of SiNTs is strongly dependent on shell thickness and crystallinity [11,19]. Presumably due to high surface area, SiNTs with thinner walls expose more reactive surface species, thereby dissolving faster than relatively thicker ones. For unannealed SiNTs, while some variation in sample-dependent dissolution kinetics exist for the 10 nm wall porous SiNTs (70–100% dissolution after 2 days), resorption of SiNTs with a thicker wall is significantly slower (38 nm: ~15%; 80 nm: ~5%) (Figure 3a) [11,19]. Interestingly, dissolution behavior of porous SiNTs is similar to bioactive anodized mesoporous Si, where the degradation occurs in the form of soluble Si(OH_)4_ (and is eliminated from the body via the kidneys in a non-toxic manner), thereby implying favorable biodegradability of this type of SiNT [1]. In contrast, within the same time frame, dissolution of the annealed SiNTs drops to less than 5% for all shell thickness; nevertheless, dissolution kinetics still follows the same trend observed in unannealed samples with thin, porous SiNTs resorbing faster than the 38 nm wall, followed by 80 nm walled SiNTs (Figure 3b).

Since dissolution behavior of SiNTs depends on the media utilized in a given experiment [11], in another experiment the complete cell culture medium was chosen since it more closely mimics biological conditions [18]. In addition, to examine the impact of the degraded byproducts of SiNTs to cell viability, human embryonic kidney (HEK) 293 cells grown in the culture medium were exposed to SiNTs. Surprisingly, while a significant amount of SiNTs with a ~50 nm wall remained visible after a 2-day incubation in PBS, SiNTs resorbed significantly faster in the complete medium (no cells), since almost all the nanotubes dissolved within the same time interval [18]. However, due to the complex nature of the growth medium, it is still unclear which compositions facilitate rapid dissolution of SiNTs. When SiNTs were incubated with HEK 293 cells, not only the dissolution of SiNTs was relatively fast, as indicated above, noticeably, cells were still healthy and proliferated normally, hence confirming biocompatibility of this material [18].

**Figure 4:**
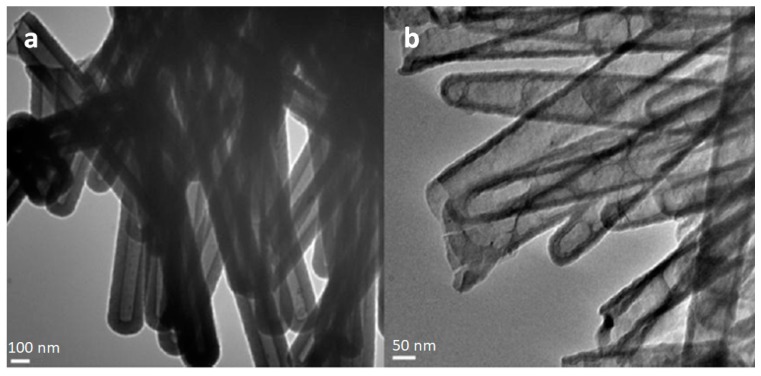
TEM images of SiNTs (**a**) before (52.38 ± 7.04 nm) and (**b**) after 3-day incubation (12.63 ± 2.83 nm) (adapted from [18] with permission).

During the course of dissolution studies using the cell culture medium, morphological changes of SiNTs in the presence of cells were also monitored. Optical imaging showed that the color of SiNT arrays transformed from opaque black (in the initial 50 nm wall thickness) to transparent brown as the Si shell gradually became thinner, reaching a value of 12.63 ± 2.83 nm after 3 days, thereby suggesting approximately 75% of SiNTs dissolved (Figure 4). However, since multiple components of the complete medium interfere with the molybdate assay, specific quantification of the dissolution rate of SiNTs in the growth medium cannot be determined via this route.

## 4. Nanotube Surface Modification Strategies Relevant to Therapeutic Applications

The versatile surface chemistry of nanostructured pSi enables attachment of numerous molecules of interest via straightforward coupling chemistry, thereby extending their utility in bio-relevant applications [17,20]. With regard to SiNTs, the native oxide surface of as-prepared material allows facile formation of stable siloxane linkages, similar to the case of oxidized pSi surfaces demonstrated previously [17,21]. Among possible modifying agents, organoalkoxysilane molecules have been widely used to alter surface properties of the materials via the introduction of a useful functional group at the other end of the organosilane species (amine, thiol, etc.) [22,23]. Much of our emphasis to date has entailed functionalization of SiNTs with amino organosilane (3-aminopropyltriethoxysilane (APTES)) to graft amino terminal groups on the SiNT surface, thereby allowing conjugation with additional molecular entities via covalent bonds (polyethylene glycol (PEG) and fluorescent dyes) or electrostatic interactions (polynucleotides). We subsequently discuss the implications of adding these specific moieties for its use as a therapeutic vehicle. Furthermore, we also demonstrate the fact that such amino-terminated SiNTs can be exploited as a synthon for formation of a dispersed scaffold of metallic nanocrystals, such as platinum (Pt), the latter of which exhibit intrinsic anti-cancer activity, thereby expanding the utility of this type of surface-modified nanostructure.

### 4.1. PEGylation of SiNTs

In addition to a lack of toxicity, nanostructures seeking use in a biological context must also remain stable in aqueous solution for long-term storage [24,25,26,27]. Depending on solution ionic strength, pH, and physicochemical properties of the nanoparticles, the extent of aggregation will vary [28]. For oxide-terminated nanoparticles, aggregation is often observed due to high surface energy of the materials [29]. One well-studied strategy to prevent particle–particle interaction is shielding the surface with hydrophilic molecules of tunable chain length, such as polyethylene glycol (PEG) [28]. In this case, PEGylation or PEG coating creates a hydrated shell surrounding the nanoparticle core, thereby sterically hindering nanoparticles from interacting with each other [30]. Besides dispersion enhancement, several studies have indicated other benefits of PEGylation, which involve the extension of systematic circulation time and reducing immunogenicity of nanoparticles in vivo [31,32].

**Figure 5:**
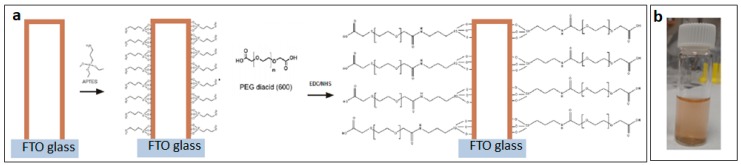
(**a**) 3-aminopropyltriethoxysilane (APTES) functionalization and PEGylation schemes of SiNTs; (**b**) Soluble SiNTs (10 nm wall thickness) in deionized water at room temperature after coating with PEG-diacid (600).

This concept was successfully demonstrated using PEG-diacid (600) moieties grafted on amino-terminated SiNT surfaces via 1-ethyl-3-(3-dimethylaminopropyl) carbodiimide (EDC) and N-hydroxysuccinimide (NHS) coupling chemistry [33]. While unmodified SiNTs tend to aggregate within a few hours after sonication, PEGylated SiNTs were not only highly dispersed in deionized H_2_O, but also remain suspended in solution for months at room temperature (Figure 5).

### 4.2. Fluorescently-Tagged SiNTs

Fluorescence-based imaging is one of the most important tools in biological studies [34]. In the specific case of gene/drug delivery using nanoparticles, it is crucial to distinguish the drug delivery vehicles from cellular components within the biological microenvironment, thereby determining the fate of the drug carrier [35]. In this regard, simple fluorophore tagging can endow non-fluorescent nanoparticles with an emissive fluorescence feature for bioimaging and related applications [36,37].

**Figure 6:**
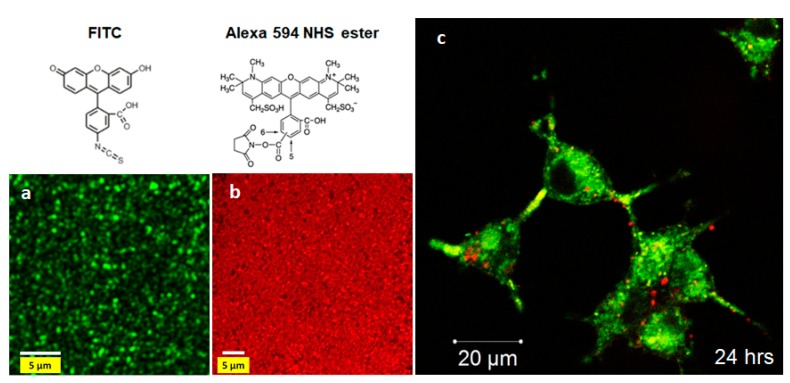
Confocal images of SiNTs after conjugated to (**a**) fluorescein isothiocyanate (FITC) and (**b**) Alexa 594 NHS ester using APTES as the coupling agent. (**c**) Cellular uptake of Alexa-labeled SiNTs (35 nm wall thickness) in human embryonic kidney (HEK) 293 cells (cytoplasm stained SP-DIOC_18_(3)) after 24 h (adapted from [18] with permission).

In this section, we evaluate the use of SiNTs terminated with THE amino group as a platform for conjugating with two different fluorescent dyes: Fluorescein isothiocyanate (FITC) (green fluorescence, λ_em_ = 520 nm) [38] and Alexa Fluor 594 NHS (succinimidyl ester) (red fluorescence, λ_em_ = 615 nm) [39]. The amine moeities of APTES molecules conjugated to the SiNT surface react with FITC and Alexa dyes to produce an isothiourea linkage and an amide bond, respectively. In both cases, APTES serves as an efficient linker to stably incorporate fluorescent dyes to the SiNT matrix. Based on confocal fluorescence imaging, SiNT surfaces were successfully labeled with FITC and Alexa dyes, as indicated in uniform emission from the nanotube arrays (Figure 6a,b) [18], therefore implying possible uses of fluorescently-labeled SiNTs in biological studies. To demonstrate this concept, Alexa-labeled SiNTs with 35 nm Si shell thickness were utilized as fluorescent probes to ideally track cellular uptake of SiNTs in HEK 293 cells. By staining the cytoplasm of HEK 293 cells with 3,3′-dioctadecyl-5,5′-di(4-sulfophenyl)oxacarbocyanine, sodium salt (SP-DIOC_18_)(3) (green fluorescence, λ_em_ = 513 nm), the interaction between Alexa-labeled SiNTs and the cells was clearly observed. Clear accumulation of the labeled SiNTs in the cytoplasm of HEK 293 cells was observed after 24-h exposure (Figure 6), thereby confirming the role of Alexa-labeled SiNTs as an option in cellular labeling [18].

### 4.3. DNA Immobilization on SiNT Surface through Electrostatic Interaction

As one of the most exciting new options in the treatment of disease, gene therapy has demonstrated extraordinary potential in the possible treatment of a variety of diseases, such as cancer and heart disease, as well as tissue repair and regeneration [40,41,42]. This process involves introducing exogenous nucleic acids (e.g., DNA plasmid and small interference RNA) into cellular compartments of the host cells [40]. In order to achieve efficient expression of the foreign genes, fragile genetic materials must be protected from degradation in a biological environment [42].

While selected viruses have been demonstrated as efficient vectors to effectively transfect cells, immunogenicity is a main concern that restricts the application of this route [43,44]. An alternative safer method is using positively charged polymers or cationic lipids to encapsulate negatively charged suitable nucleic acids via electrostatic interactions, thereby forming a stable complex while protecting the cargos from degradation [45,46]. Inorganic nanoparticles are yet another appealing option in this regard, owing to their tunable structures, surface chemistry, and compositions [47,48].

**Figure 7:**
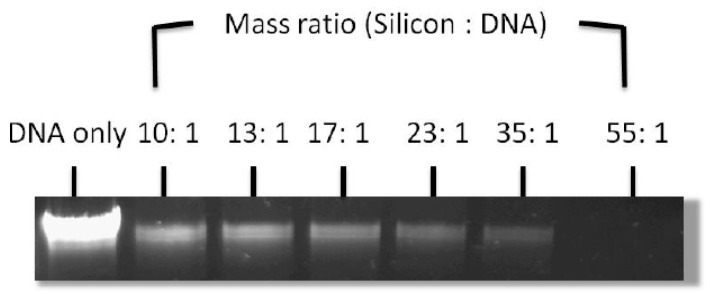
Agarose gel electrophoresis assay showing the unbound plasmid DNA (pDNA) remaining in the solution after incubation with functionalized SiNTs at varying mass ratio (APTES-SiNTs: pDNA). The band intensity reduces as the mass ratio increases, thereby indicating more pDNA bound to the functionalized SiNTs (adapted from [18] with permission).

Along these lines, we have demonstrated the use of SiNTs as a potential carrier to encapsulate and deliver genetic materials, specifically plasmid DNA (pDNA) encoding green fluorescent protein (GFP) [18]. Initial grafting with APTES (pKa = 9.6) converts the negative surface charge of SiNTs to positive owing to the presence of the amino groups, thereby enabling immobilization of pDNA on the SiNT surface [49]. Formation of pDNA/APTES-SiNTs complexes was evaluated by determining the amount of pDNA remaining in the supernatant by an agarose gell electrophoresis assay. Our results indicate complete binding of pDNA to functionalized SiNTs at a 55:1 (pDNA:SiNTs) mass ratio, thereby suggesting potential use of SiNTs as a vector in gene therapy (Figure 7). Evaluation of transfection efficiencies using this system in a suitable in vitro model are underway.

### 4.4. Functionalized SiNTs as a Template for Formation of Platinum Nanocrystals

Since FDA approval in 1978, the platinum-based drug cisplatin has been effectively utilized to treat a variety of cancers (lymphomas, carcinoma, etc.) [50,51,52]. However, a lack of specificity and selectivity has led to multiple detrimental side effects, thereby raising legitimate caution in its use as a chemotherapeutic agent [51]. In order to improve the therapeutic efficiency of cisplatin, several drug carrier systems have been developed, such as polymers or inorganic nanoparticles, to effectively deliver a desired payload while avoiding nonspecific delivery to healthy cells [50,53,54,55].

In terms of possible carrier candidates, SiNTs with ample interior space can ideally load therapeutic molecules, while surface modification of the nanotube carrier allows possible targeting ability for delivery and subsequent release of a useful drug at the disease site(s). One initially envisioned approach involves cisplatin attachment to SiNTs via a linker strategy, in which amino moieties of APTES coordinate to Pt complexes; a locally-concentrated amount of the drug is thereby clustered on SiNTs and, if coupled with a suitably-functionalized targeting peptide (or the alternative) present on the nanotube surface, is ideally delivered to cancer cells.

**Figure 8:**
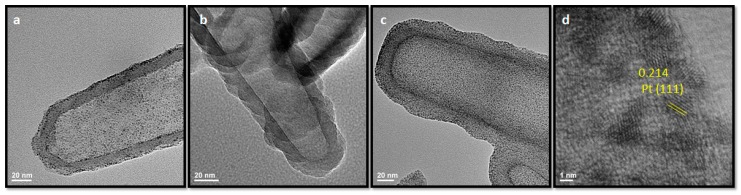
TEM images of SiNTs after incubated in (**a**) impure cisplatin (~15 wt % platinum (Pt), evaluated by erergy dispersive x-ray analysis (EDX), (**b**) pure cisplatin (>95%) (~2 wt % Pt, EDX), (**c**) K_2_PtCl_4_ (50 wt % Pt, EDX)_._ (**d**) High resolution TEM image showing lattice spacing of Pt species deposited on SiNTs after using K_2_PtCl_4_ as a Pt precursor.

Interestingly, in initial experiments, instead of observing the intact molecular Pt complexes adsorbed on the SiNT surface, we detected a highly dense cluster of crystalline platinum species (1–3 nm) uniformly deposited on SiNTs (Figure 8a). By evaluating possible impurities in the cisplatin using standard spectrometric assays of the λ_310_/λ_247_ ratio [56], we discovered the presence of significant amounts of K_2_PtCl_4,_ (common precursor in cisplatin synthesis) and that this species was responsible for the formation of elemental Pt on functionalized SiNTs [57]. As Pt nanocrystals (Pt NCs) preferentially form on functionalized SiNTs, but not on as-prepared unmodified SiNTs, it is clear that the presence of the primary amine moieties of APTES coordinate with PtCl_4_^2-^ and play a role in the reduction of Pt^2+^ to Pt^0^.[58]

Although this result is unexpected in terms of cisplatin loading, we have discovered a new synthetic route to readily synthesize ultra-small Pt NCs on the SiNT matrix using K_2_PtCl_4_ as a Pt precursor. While Pt-based drugs (e.g., cisplatin, carboplatin) have been exploited in cancer treatment, noticeably, reports in recent years have demonstrated Pt NCs (1–3 nm) as an alternative anticancer reagent which can effectively overcome chemoresistance in some cancer cell lines, such as hepatocellular carcinoma (HCC) [59,60,61]. Inspired by these preceding studies, investigations in Pt NCs deposition on SiNTs using K_2_PtCl_4_ and in vitro toxicity of the Pt NCs-SiNT composites are ongoing in our lab to evaluate the associated therapeutic activity of this novel material.

## 5. Loading of the Nanotube Interior

**Figure 9:**
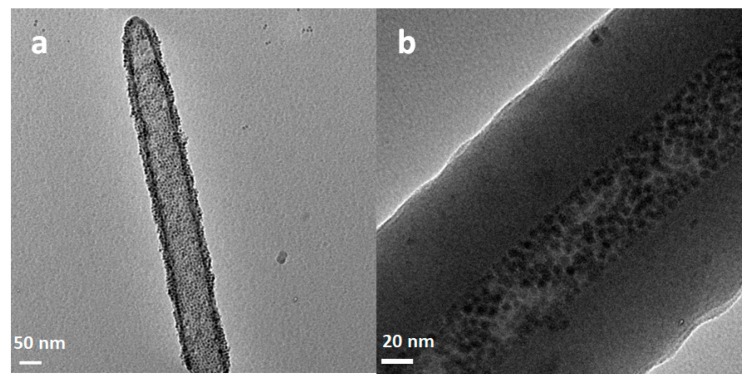
TEM images of (**a**) 4 nm Fe_3_O_4_ nanocrystals loaded into 10 nm shell SiNTs; (**b**) 4 nm Fe_3_O_4_ nanocrystals loaded in 70 nm thick SiNTs.

From a geometric perspective, the rather large and tunable inner cavity of a SiNT presents a significant loading opportunity for applications involving delivery of a therapeutic cargo. In a rather novel twist to this strategy, we have actually demonstrated the efficient loading of biocompatible superparamagnetic iron oxide nanoparticles into the interior of different SiNT inner diameters (Figure 9) [62,63].

Using this strategy, one can ideally achieve targeted delivery of a drug attached to the outer nanotube surface, while guided by the presence of an external magnetic field to the desired site in vitro/in vivo [64]. This is in contrast to the alternative strategy of functionalizing the outer nanotube surface with a targeting moiety (e.g., antibody or peptide) and subsequently loading the nanotube interior with a desired therapeutic species.

## 6. Conclusion

This review has covered a number of key aspects of SiNTs that have interesting implications in therapeutics. While additional opportunities remain, multiple clear advantages of exploiting surface tunability of SiNTs for exploiting their functional tubular structure have been demonstrated. Studies involving biocompatibility of the SiNTs and applications in drug/gene delivery are underway to broaden our knowledge of its interactions with biological systems and potential applications of this novel one-dimensional material.
